# Recent changes in trends of opioid overdose deaths in North America

**DOI:** 10.1186/s13011-020-00308-z

**Published:** 2020-08-31

**Authors:** Sameer Imtiaz, Kevin D. Shield, Benedikt Fischer, Tara Elton-Marshall, Bundit Sornpaisarn, Charlotte Probst, Jürgen Rehm

**Affiliations:** 1 Institute for Mental Health Policy Research, Centre for Addiction and Mental Health, 33 Russell Street, Toronto, ON M5S 2S1 Canada; 2 Dalla Lana School of Public Health, University of Toronto, 6th Floor, 155 College Street, Toronto, ON M5T 3M7 Canada; 3 Institute of Medical Science, University of Toronto, Room 2374, 1 King’s College Circle, Toronto, ON M5S 1A8 Canada; 4 Department of Psychiatry, University of Toronto, 8th Floor, 250 College Street, Toronto, ON M5T 1R8 Canada; 5 Schools of Population Health and Pharmacy, Faculty of Medical and Health Sciences, University of Auckland, 85 Park Road, Grafton, Auckland, 1023 New Zealand; 6 Centre for Applied Research in Mental Health & Addiction, Faculty of Health Sciences, Simon Fraser University, 515 W. Hastings Street, Vancouver, V6B 5K3 Canada; 7 Ontario Tobacco Research Unit, Centre for Addiction and Mental Health, 33 Russell Street, Toronto, ON M5S 2S1 Canada; 8 Department of Epidemiology and Biostatistics, Schulich School of Medicine and Dentistry, Western University, Kresge Building, 1151 Richmond Street, London, ON N6A 5C1 Canada; 9 Campbell Family Mental Health Research Institute, Centre for Addiction and Mental Health, 250 College Street, Toronto, ON M5T 1R8 Canada; 10 Institute for Clinical Psychology and Psychotherapy, TU Dresden, Chemnitzer Str. 46, 01187, Dresden, Germany; 11 Department of International Health Projects, Institute for Leadership and Health Management, I.M. Sechenov First Moscow State Medical University, Trubetskaya Str., 8, B. 2, Moscow, 119992 Russian Federation

**Keywords:** Analgesics, opioid, Prescription drug, Death, Drug overdose, United States, Canada, Ontario, British Columbia

## Abstract

**Background:**

As several regulatory and environmental changes have occurred in North America, trends in overdose deaths were examined in the United States (US), Ontario and British Columbia (BC), including changes in consumption levels of prescription opioids (PO) and overdose deaths, changes in correlations between consumption levels of PO and overdose deaths and modeled differences between observed and predicted overdose deaths if no changes had occurred.

**Methods:**

Consumption levels of PO included defined daily doses for statistical purposes per million inhabitants per day for the US and Canada (2001–2015). Overdose deaths included opioid overdose deaths for the US (2001–2017) and Ontario (2003–2017) and illicit drug overdose deaths for BC (2001–2017). The analytic techniques included structural break point analyses, Pearson product-moment correlations and multivariate Gaussian state space modeling.

**Results:**

Consumption levels of PO changed in the US in 2010 and in Canada in 2012. Overdose deaths changed in the US in 2014 and in Ontario and BC in 2015. Prior to the observed changes in consumption levels of PO, there were positive correlations between consumption levels of PO and overdose deaths in the US (*r* = 0.99, *p* < 0.001) and Ontario (*r* = 0.92, *p* = 0.003). After the observed changes in consumption levels of PO, there was a negative correlation between consumption levels of PO and overdose deaths in the US (*r* = − 0.99, *p* = 0.002). Observed overdose deaths exceeded predicted overdose deaths by 5.7 (95% Confidence Interval [CI]: 4.8–6.6), 3.5 (95% CI: 3.2–3.8) and 21.8 (95% CI: 18.6–24.9) deaths per 100,000 people in the US, Ontario and BC, respectively in 2017. These excess deaths corresponded to 37.7% (95% CI: 31.9–43.6), 39.2% (95% CI: 36.3–42.1) and 72.2% (95% CI: 61.8–82.6) of observed overdose deaths in the US, Ontario and BC, respectively in 2017.

**Conclusions:**

The opioid crisis has evolved in North America, as a sizeable proportion of overdose deaths are now attributable to the several regulatory and environmental changes. These findings necessitate substance use policies to be conceptualized more broadly as well as the continued expansion of harm reduction services and types of pharmacotherapy interventions.

## Introduction

Opioid overdose deaths represent a significant public health crisis in North America. Parallel to the rise in prescription opioid use and non-medical prescription opioid use, overdose deaths in North America have reached unprecedented levels over the past two decades [1–3]. These deaths increased steadily in the United States [4, 5] and the Canadian province of Ontario [6, 7]. They remained stable in the Canadian province of British Columbia from 2004 to 2013, with substantial increases from then onwards [8]. Opioid overdoses were responsible for 49,068 deaths in the United States in 2017, constituting the majority of illicit drug overdose deaths (67.8%), far exceeding motor vehicle injury deaths (37,133 deaths), previously the largest cause of unintentional injury deaths [9]. Although comprehensive national data are not available, opioid overdoses resulted in more than 4000 deaths in Canada in 2017 [2]. Overdose deaths are now one of the main causes of the recent decrease in life expectancies in the United States [10–12]. A plateauing of life expectancy has been documented in Canada. However, opioid overdose deaths are partly responsible for decreases in life expectancies observed in British Columbia from 2014 to 2016 [13].

One of the primary drivers of opioid overdose deaths has been opioid prescribing. At the population level, it has been demonstrated based on data from the past decade that there are strong temporal correlations between opioid prescribing - overall and formulation-specific - and opioid-related harms, including opioid overdose deaths [14–16]. Recent evidence suggests that although opioid overdose deaths have been increasing in the United States, opioid prescribing has declined [17]. Indeed, the diversion and misuse of prescription opioids plateaued or declined in the United States between 2011 and 2013 [5]. Similar declines in opioid prescribing are also evident in Canada [18]. These declines may be attributable to the recent introduction of co-occurring regulatory changes, including, but not limited to, the implementation of prescription-monitoring programs [19, 20] and crackdowns on inappropriate prescription dispensation (e.g. pill mills) [21], as well as restrictions on opioid marketing [22] and opioid prescribing [23–25] (including the development of prescription guidelines) [26, 27].

In addition to the abovementioned regulatory changes, other environmental changes may have more recently impacted opioid overdose deaths as well, including decreases in the availability of prescription opioids and cost of illicit opioids. Although fentanyl is a prescription drug [28], illicit fentanyl and fentanyl analogs are also commonly available [29, 30]; however, toxicological analyses of opioid overdose deaths are often not able to distinguish between prescription fentanyl and illicit fentanyl [29, 31]. It is suggested that the main driver of opioid overdose deaths more recently in the United States and Canada is illicit opioids, including illicit fentanyl [1, 3, 32, 33] and heroin [34], unlike the period prior to the regulatory changes [35, 36]. At the same time, health authorities have implemented and scaled up a variety of harm reduction interventions in the United States and Canada, including supervised consumption facilities, distribution of naloxone kits and expansion of addiction pharmacotherapy [37].

However, the overall impact of these regulatory and environmental changes on opioid overdose deaths is not well characterized. As such, we analyzed recent data from the United States, Ontario and British Columbia to examine trends in opioid overdose deaths to inform the public health response. Our specific objectives were as follows,

1. Examine changes in consumption levels of prescription opioids and overdose deaths.
2. Assess changes in the correlations between consumption levels of prescription opioids and overdose deaths.
3. Compare predicted overdose deaths with observed overdose deaths if no regulatory or environmental changes had occurred.

## Methods

### Data sources

National data on consumption levels of prescription opioids in the United States and Canada (2001 to 2015) were obtained in the form of defined daily doses for statistical purposes per million inhabitants per day (S-DDD) from the International Narcotics Control Board (see [28] for the last iteration of the report). This metric facilitates comparisons between different kinds of opioids (including codeine, fentanyl, hydrocodone, hydromorphone, methadone, morphine, oxycodone, pethidine, tilidine, others) based on their potency, which is computed by divisions of the annual consumption of prescription opioids by 365, population (millions) during the year and defined daily dose. Importantly, this metric captures the total availability or volume of prescription opioids in the population. As this metric is reported according to three-year moving average intervals, annual estimates were ascertained using the midpoint of a three-year moving average (e.g., moving average for 2014 to 2016 was used for 2015).

Consistent with the definition of the Centers for Disease Control and Prevention [38], opioid overdose deaths in the United States (2001 to 2017) included drug overdose deaths (International Classification of Diseases, Tenth Revision (ICD-10) X40–44, X60–64, X85 and Y10–14) that involved opioids (ICD-10 T40.0–40.4 and T40.6) as a contributory cause [38], which were obtained from the United States National Vital Statistics System Multiple Cause-of-Death Files [39]. Contrastingly, national time series on opioid overdose deaths are not available in Canada. In addition, case definitions of opioid overdose deaths often differ between provinces. Therefore, Ontario and British Columbia were examined, two provinces representing about half of the Canadian population, who experienced the largest number of opioid overdose deaths in 2017 [2]. Based on the definition of the Office of the Chief Coroner for Ontario [40], opioid overdose deaths in Ontario (2003 to 2017) included all deaths where opioid overdose was considered to be a contributor to the cause of death (including intentional deaths). On the other hand, unintentional illicit drug overdose deaths served as an approximation of opioid overdose deaths in British Columbia (2001 to 2017) [41]. Based on the definition of the British Columbia Coroners Service, illicit drug overdose deaths included accidental and undetermined deaths that involved illicit drugs and unprescribed medications, as well as their combinations with other prescribed medications [41]. We note that while the vast majority of illicit drug overdose deaths involved opioids, non-opioid overdose deaths (e.g., cocaine and methamphetamine) were included as well.

All data on consumption levels of prescription opioids and overdose deaths are presented in Table S1 in the Supplementary Appendix.

### Statistical analyses

To test for changes over time in consumption levels of prescription opioids and overdose deaths, supremum Wald tests were conducted, which search for the maximum of the sample individual Wald tests constructed by testing each possible break date in the sample [42, 43]. A symmetric data trimming of 15% was performed to avoid testing for break points that were too close to the beginning or end of the time series [42]. The trends in consumption levels of prescription opioids and overdose deaths were characterized separately for the periods before and after the break points of each jurisdiction.

Correlations between the consumption levels of prescription opioids and overdose deaths were quantified using Pearson’s product-moment correlations. For each computed correlation, 95% Confidence Intervals (CIs) and *P* values were also generated. These correlations were calculated separately for the periods before and after changes in consumption levels of prescription opioids in each jurisdiction, as determined by the abovementioned break point analyses.

Overdose deaths assuming no significant regulatory or environmental changes were modeled to determine the extent the observed deaths differed from predicted deaths based on prior trends. Predicted overdose deaths were modelled using multivariate Gaussian state space models [44]. No restrictions were assumed for the covariance structures of the level or noise components. Initial estimates were calculated according to the covariance matrix of the observed series in keeping with the procedures outlined by Helske [45]. These initial values were used in subsequent estimation procedures, which determined final model parameters through importance sampling (using 1000 samples) and a Broyden–Fletcher–Goldfarb–Shanno algorithm for likelihood computation. The smoothed estimates of states were generated using Kalman filtering and smoothing, with exact diffuse initialization using a univariate approach for exponential family state space models. Recursive residuals from the models were inspected to check whether autocorrelation remained in the residuals.

All analyses were performed using the statistical software R version 3.5.1 and the statistical software package KFAS for R [45, 46].

### Ethics compliance

Research ethics committee review and approval were not required, as aggregate data were extracted from publicly available sources.

## Results

### Trends in consumption levels of prescription opioids and overdose deaths

**Fig. 1:**
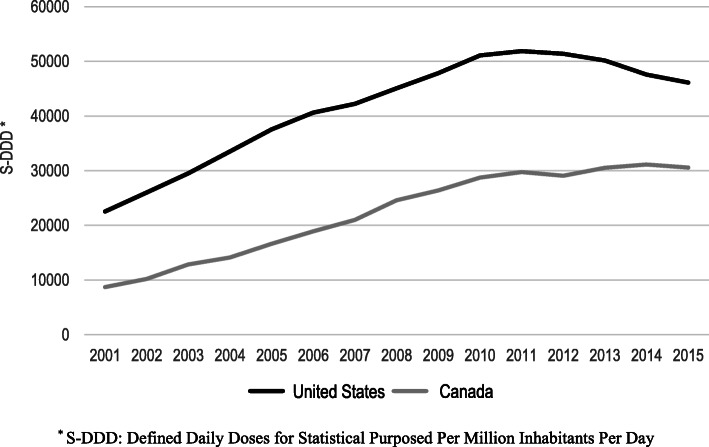
Defined daily doses for statistical purposes per million inhabitants per day of prescription opioids

Consumption levels of prescription opioids increased by 130.3% in the United States from 2001 to 2011 (22,524 to 51,873 S-DDD) (Fig. 1). The consumption levels of prescription opioids in the United States changed in 2012, decreasing by 10.3% from 2012 to 2015 (51,374 to 46,090 S-DDD). Similar changes occurred in Canada in 2010. Consumption levels of prescription opioids increased by 202.8% from 2001 to 2009 (8713 to 26,380 S-DDD) and increased by 6.4% from 2010 to 2015 (28,731 to 30,570 S-DDD).

Overdose deaths changed in the United States in 2014 (Table 1). After increasing by 138.2% from 2001 to 2013, overdose deaths increased by 67.7% from 2014 to 2017. Similar changes occurred in both Ontario and British Columbia in 2015. Overdose deaths in Ontario increased by 65.3% from 2003 to 2014 and by 68.8% from 2015 to 2017. On the other hand, overdose deaths in British Columbia increased by 36.8% from 2001 to 2014 and by 169.0% from 2015 to 2017.

**Table 1:** Changes in overdose deaths

Country and province	Time period	Overdose deaths^**a**^			
		From (Per 100,000 people)	To (Per 100,000 people)	Percentage change (%)	Annualized Change (Per 100,000 people per year)
Canada					
Ontario					
	2003–2014	3.0	4.9	65.3%	0.2
	2015–2017	5.3	8.9	68.8%	1.3
British Columbia					
	2001–2014	5.8	7.9	36.8%	0.2
	2015–2017	11.2	30.1	169.0%	7.4
United States					
	2001–2013	3.3	7.9	138.2%	0.4
	2014–2017	9.0	15.1	67.7%	1.8

^a^As determined by the break point analyses, overdose deaths changed in Ontario in 2015, in British Columbia in 2015 and in the United States in 2014

### Correlations between consumption levels of prescription opioids and overdose deaths

Prior to the observed changes in consumption levels of prescription opioids, consumption levels of prescription opioids and overdose deaths were positively correlated in the United States (R: 0.989; 95% CI: 0.957 to 0.997) and Ontario (R: 0.922; 95% CI: 0.553 to 0.988) (Table 2). After the observed changes in consumption levels of prescription opioids, consumption levels of prescription opioids and overdose deaths were negatively correlated in the United States (R: -0.985, 95% CI: − 1.00 to − 0.442), not Ontario. Such correlations were not observed during either period in British Columbia.

**Table 2:** Correlations between defined daily doses for statistical purposes per million inhabitants per day of prescription opioids and overdose deaths

Country and province	Data availability	Pearson correlation coefficient: pre-change in S-DDD of prescription opioids^**a**^			Pearson correlation coefficient: post change in S-DDD of prescription opioids^**b**^		
		Estimate	(95% CIs)	***P*** value	Estimate	(95% CIs)	***P*** value
Canada							
Ontario^c,d^	2003–2015	0.922	(0.553, 0.988)	0.003	0.760	(−0.135, 0.972)	0.080
British Columbia^c,e^	2001–2015	−0.288	(−0.799, 0.465)	0.452	0.730	(−0.200, 0.968)	0.100
United States^d^	2001–2015	0.989	(0.957, 0.997)	< 0.001	−0.985	(−1.00, − 0.442)	0.002

^a^Pre-change in consumption levels of prescription opioids was 2001 to 2011 in the United States, 2003 to 2009 in Ontario and 2001 to 2009 in British Columbia, as determined by the break point analyses

^b^Post change in consumption levels of prescription opioids was 2012 to 2015 in United States, 2010 to 2015 in Ontario and 2010 to 2015 in British Columbia, as determined by the break point analyses

^c^Defined daily doses data for statistical purposes per million inhabitants per day are not province-specific

^d^Overdose deaths are due to opioid use

^e^Overdose deaths are due to illicit drug use

### Predicted overdose deaths compared to observed overdose deaths

**Fig. 2:**
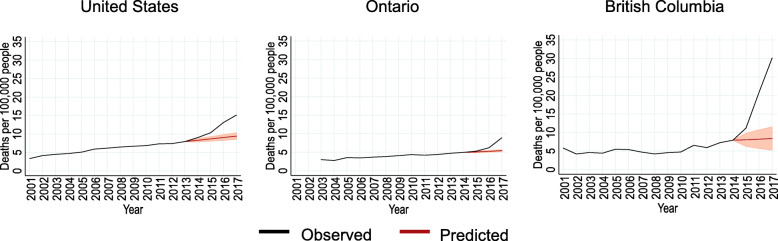
Observed and predicted overdose deaths assuming no regulatory or environmental changes

Based on the period before the changes in overdose deaths, overdose deaths were predicted for all three jurisdictions (Fig. 2). If overdose deaths had continued to increase at rates observed before the changes, 9.4 (95% CI: 8.5 to 10.2), 5.1 (95% CI: 4.9 to 5.3) and 8.4 (95% CI: 5.2 to 11.5) overdose deaths per 100,000 people were predicted for the United States, Ontario and British Columbia in 2017, respectively. The observed overdose deaths exceeded the predicted overdose deaths by 5.7 (95% CI: 4.8 to 6.6), 3.5 (95% CI: 3.2 to 3.8) and 21.8 (95% CI: 18.6 to 24.9) deaths per 100,000 people in the United States, Ontario and British Columbia in 2017, respectively. The differences corresponded to 37.7% (95% CI: 31.9 to 43.6%), 39.2% (95% CI: 36.3 to 42.1%) and 72.2% (95% CI: 61.8 to 82.6%) of observed overdose deaths in the United States, Ontario and British Columbia in 2017, respectively.

## Discussion

Consumption levels of prescription opioids have decreased in the United States since 2012 and increased slightly in Canada since 2010. In agreement with findings from our present analyses, others have also demonstrated reductions in morphine milligram equivalents per capita since 2010 and opioid prescription rates per capita since 2012 in the United States [17]. The slight variation in findings is attributable to selection of outcome metrics and analytical strategies. Similar decreases are also evident in Canada since 2012, although comprehensive time series are not available [18, 47].

Overdose deaths have increased in the United States since 2014 and in Ontario and British Columbia since 2015. Prior to changes in the consumption levels of prescription opioids in the United States and Ontario, consumption levels of prescription opioids were positively correlated with overdose deaths. However, consumption levels of prescription opioids were negatively correlated with overdose deaths in the United States after 2012; as consumption levels of prescription opioids decreased, overdose deaths increased. Consumption levels of prescription opioids are no longer associated with overdose deaths in Ontario, while they were not associated with overdose deaths during the pre-change or post-change periods in British Columbia.

Overdose deaths have increased at a greater rate than would be expected in the absence of regulatory and environmental changes in the United States since 2014 and in Ontario and British Columbia since 2015. Our modeling predictions are corroborated by another examination of opioid-related deaths in Ontario from 2013 to 2016, where 39.8% of deaths were documented to have occurred among those without an active opioid prescription [4]. Almost identical findings were obtained from our present analyses, as 39.2% of overdose deaths were attributable to regulatory and environmental changes in Ontario in 2017.

Decreases in consumption levels of prescription opioids reflect changes in opioid prescribing practices. The reductions in morphine milligram equivalents per capita in the United States highlighted above stem from reductions in opioid prescriptions and doses per opioid prescription [17]. However, as the duration of opioid prescriptions has increased at the same time in the United States, it is probable that opioid therapy initiation is occurring less often, but those receiving opioids are likely to continue receiving them [17]. Although comprehensive time series are not available for all of Canada, similar trends have been noted based on recent observations from select provinces, which indicate reductions in opioid prescriptions (overall and initiations), as well as reductions in long-term opioid prescriptions and doses per opioid prescription for long-term prescriptions [47].

Changes in the correlations between consumption levels of prescription opioids and overdose deaths in the United States and Ontario likely stem from the recent regulatory and environmental changes. Illicit opioids have often replaced prescription opioids as the substance of choice (see for the transition from prescription opioids to heroin in general: [48–50]; for illicit fentanyl: [51]). Importantly, other illegal substances often include added fentanyl because of its availability and cheap price [52]. Consistent with transitions in the types of opioids, state adoption of prescription-monitoring programs intended to curtail inappropriate opioid prescribing has been shown to be associated with steadily increasing heroin overdose deaths in the United States [53]. The null correlations observed for British Columbia may be explained by a different outcome metric in illicit drug overdose deaths, as well as a different drug environment that features greater availability of illicit opioids, given the geographical proximity to countries where such substance originate [31].

The comparison of predicted and observed overdose deaths underscores jurisdictional differences in the impacts of regulatory and environmental changes. About 40% of overdose deaths in the United States and Ontario were attributable to these recent changes in 2017. The situation was drastically different in British Columbia, where more than 70% of overdose deaths were attributable to these recent changes in 2017. More recent observations indicate that the differences between the jurisdictions may be narrowing as well. Indeed, opioid overdose deaths involving synthetic opioids (including fentanyl) increased in the United States from 46 to 60% between 2016 and 2017 [54]. On that same note, unintentional opioid overdose deaths involving fentanyl increased from 45 to 69% in Ontario and from 69 to 83% in British Columbia between 2016 and 2017 [2].

Although overdose deaths are typically classified statistically as being due to one substance in toxicological analyses, they are often caused by the adverse and interactive effects of multiple substances [55]. These interactions may involve different opioids (e.g., prescription opioids combined with other illegal opioids) with additive effects, or combinations with sedatives (e.g., benzodiazepines and alcohol), all of which have been identified as major contributors to overdose deaths [6, 49, 56, 57]. An analysis of 13 states in the United States demonstrated that alcohol was involved in 18.5% of prescription opioid-related emergency visits and 22.1% of prescription opioid-related deaths [58]. On a similar note, about 75% of opioid overdose deaths involve non-opioid substances in Canada [2]. Basing prevention interventions solely on the basis of such toxicological analyses is therefore somewhat misleading.

Substance use policies should be conceptualized with a broad focus [59, 60]. Reductions in opioid prescribing are needed [25], as consumption has risen sharply during the past two decades in North America [61] beyond the support of solid evidence for efficacy [62], resulting in extensive harms. However, despite the apparent need to address this problem, the potential solutions are not clear [63]. It is evident that the regulatory and environmental changes have resulted in more harms, as the supply voids created by the depletion of prescription opioids have been filled by the proliferation of illicit opioids, rendering the current crisis as one involving toxic drug exposure [63, 64]. The situation necessitates protection of the public through the provision of a safe opioid supply (see [63–65] for proposed models). Additional measures are also needed, such as expanding access to different types of opioid use disorder pharmacotherapy (e.g. morphine, hydromorphone) [66], as well as scaling-up harm reduction interventions to prevent overdose deaths, including the distribution of Naloxone and implementation of supervised injection facilities (for overviews, see [67–69]).

### Study limitations

There are some limitations that should be considered in the interpretation of the findings. First, the classification of overdose deaths differed between the three examined jurisdictions. The main difference pertained to the inclusion of intentional injury, with intentional overdoses included in the United States and Ontario, unlike British Columbia. In addition, there were differences between the United States and Ontario and British Columbia in terms of the inclusion of overdose deaths involving illicit drugs other than opioids. Regardless of the definitions used, unintentional overdose deaths are often hard to differentiate from intentional overdose deaths, thereby creating additional measurement error [70]. On that same note, there may have been other subjective and procedural differences in the ascertainment of overdose deaths by health authorities between the three jurisdictions. Second, there may have been ascertainment differences in overdose deaths within the three jurisdictions, as the likelihood of case detection may have differed during the study period. Third, metrics that combine the use of different opioids into a single indicator rely on assumptions concerning the patterns and potency. The S-DDD operationalized in the present analyses is no exception. This metric attempts to adjust for different potencies by considering the recommended daily use of each prescription opioid, which may lead to bias (e.g., [71]). Although some bias may be included because prescription practices may differ according to world regions, it is clear that the S-DDD is best available metric for inter-jurisdictional comparisons over time. Fourth, impacts of the specific regulatory and environmental changes could not be characterized separately, given the limited points of observation (*N* = 15 for Ontario and *N* = 17 for British Columbia and United States) in between co-occurring changes. On that same note, the correlations between consumption levels of prescription opioids and overdose deaths after the observed changes in consumption levels of prescription opioids were based on four observations for the United States and six observations for Ontario and British Columbia.

## Conclusion

As the regulatory and environmental changes have occurred in North America, there have been changes in the correlations between consumption levels of prescription opioids and overdose deaths in the United States and Ontario. In all three examined jurisdictions, a sizeable proportion of overdose deaths can be attributed to these changes.

## Supplementary information

**Additional file 1:** Data on consumption levels of prescription opioids and overdose deaths.

## Data Availability

All data generated or analyzed during this study are included in this published article and its supplementary appendix.

## Abbreviations

CI: Confidence Interval
ICD-10: International Classification of Diseases, Tenth Revision
S-DDD: Defined daily doses for statistical purposes per million inhabitants per day
