# Supplementary material for: Recent changes in trends of opioid overdose deaths in North America
Source: Subst Abuse Treat Prev Policy. 2020 Aug 31;15:66. doi: 10.1186/s13011-020-00308-z (PMC7457770; doi:10.1186/s13011-020-00308-z)
Supplement: Supplementary file 1 — Additional file 1: Data on consumption levels of prescription opioids and overdose deaths. [file 13011_2020_308_MOESM1_ESM.docx]

**Supplementary Appendix To:** Recent Changes in Trends of Opioid Overdose Deaths in North America: Implications for Prevention and Policy

**Table S1: Defined daily doses of prescription opioids per million inhabitants per day and overdose deaths**

| **Year** | **Defined Daily Doses^a^** | | **Overdose Deaths** | | |
| --- | --- | --- | --- | --- | --- |
|  |  | |  | | |
|  | Canada | United States | Ontario^b^ | British Columbia^c^ | United States^b^ |
| 2001 | 8713 | 22524 |  | 236 | 9496 |
| 2002 | 10209 | 25993 |  | 172 | 11920 |
| 2003 | 12840 | 29500 | 366 | 190 | 12940 |
| 2004 | 14133 | 33532 | 340 | 183 | 13756 |
| 2005 | 16628 | 37565 | 444 | 230 | 14918 |
| 2006 | 18914 | 40604 | 436 | 229 | 17545 |
| 2007 | 20990 | 42230 | 468 | 202 | 18516 |
| 2008 | 24580 | 45054 | 491 | 183 | 19582 |
| 2009 | 26380 | 47809 | 529 | 201 | 20422 |
| 2010 | 28731 | 51081 | 571 | 211 | 21089 |
| 2011 | 29743 | 51873 | 556 | 294 | 22784 |
| 2012 | 29067 | 51374 | 585 | 269 | 23166 |
| 2013 | 30540 | 50142 | 639 | 333 | 25052 |
| 2014 | 31132 | 47580 | 676 | 368 | 28647 |
| 2015 | 30570 | 46090 | 728 | 526 | 33091 |
| 2016 |  |  | 867 | 993 | 42249 |
| 2017 |  |  | 1265 | 1452 | 49068 |

^a^ Defined daily doses data are not province-specific

^b^ Overdose deaths are defined as including intentional and unintentional deaths due to opioid use

^c^ Overdose deaths are defined as including unintentional and undetermined deaths due to illicit drug use
